# Non-destructive Plant Morphometric and Color Analyses Using an Optoelectronic 3D Color Microscope

**DOI:** 10.3389/fpls.2018.01409

**Published:** 2018-09-25

**Authors:** Hugo G. Lazcano-Ramírez, Andrea Gómez-Felipe, David Díaz-Ramírez, Yolanda Durán-Medina, Lino Sánchez-Segura, Stefan de Folter, Nayelli Marsch-Martínez

**Affiliations:** ^1^Departamento de Biotecnología y Bioquímica, Unidad Irapuato, Centro de Investigación y de Estudios Avanzados del Instituto Politécnico Nacional (CINVESTAV-IPN), Irapuato, Mexico; ^2^Unidad de Genómica Avanzada (LANGEBIO), Centro de Investigación y de Estudios Avanzados del Instituto Politécnico Nacional (CINVESTAV-IPN), Guanajuato, Mexico; ^3^Departamento de Ingeniería Genética, Unidad Irapuato, Centro de Investigación y de Estudios Avanzados del Instituto Politécnico Nacional (CINVESTAV-IPN), Irapuato, Mexico

**Keywords:** plant phenotyping, optoelectronic 3D color microscope, plant development, digital microscopy, plant morphometry, plant topology analysis, plant color analysis, Arabidopsis phenotype

## Abstract

Gene function discovery in plants, as other plant science quests, is aided by tools that image, document, and measure plant phenotypes. Tools that acquire images of plant organs and tissues at the microscopic level have evolved from qualitative documentation tools, to advanced tools where software-assisted analysis of images extracts quantitative information that allows statistical analyses. They are useful to perform morphometric studies that describe plant physical characteristics and quantify phenotypes, aiding gene function discovery. In parallel, non-destructive, versatile, robust, and user friendly technologies have also been developed for surface topography analysis and quality control in the industrial manufacture sector, such as optoelectronic three-dimensional (3D) color microscopes. These microscopes combine optical lenses, electronic image sensors, motorized stages, graphics engines, and user friendly software to allow the visualization and inspection of objects of diverse sizes and shapes from different angles. This allow the integration of different automatically obtained images along the *Z* axis of an object, into a single image with a large depth-of-field, or a 3D model in color. In this work, we explored the performance of an optoelectronic microscope to study plant morphological phenotypes and plant surfaces in different model species. Furthermore, as a “proof-of-concept,” we included the phenotypic characterization (morphometric analyses at the organ level, color, and cell size measurements) of Arabidopsis mutant leaves. We found that the microscope tested is a suitable, practical, and fast tool to routinely and precisely analyze different plant organs and tissues, producing both high-quality, sharp color images and morphometric and color data in real time. It is fully compatible with live plant tissues (no sample preparation is required) and does not require special conditions, high maintenance, nor complex training. Therefore, though barely reported in plant scientific studies, optoelectronic microscopes should emerge as convenient and useful tools for phenotypic characterization in plant sciences.

## Introduction

The plant kingdom is rich in structures, textures, shapes, and colors. Their careful study can provide valuable biological information. Efforts to document the forms available in nature, as images and morphometric information that can be mined, have impact in a wide range of research topics including, but not limited to, taxonomical classification (Cope et al., 2012) and species characterization (Pinheiro and De Barros, 2008), phenotypic variation (Passardi et al., 2007), studies at the population level (Vanderhoeven et al., 2002), and paleo-ecological studies where the analysis of the morphology of fossilized plants provides data about climate change (Chaloner and McElwain, 1997). Detailed morphometric information also helps to better understand the effect of genes or the environment on development or the interaction with other organisms. It is especially useful to characterize gene functions, when the alterations in the phenotypes of mutants are subtle, not easily detectable by simple observation. Scanning electron microscopes (SEMs) can image plant tissue and cell surfaces at high resolution. To obtain an “image,” the object is irradiated with electrons instead of photons, leading to sharp images (Smith and Oatley, 1955; Oatley et al., 1966). It has advantages over conventional optical microscopy at capturing high quality images of plant surfaces. However, the equipment (and its operation) can be expensive, and frequently, it has to be placed in conditioned rooms for optimal function, which implicate extra costs. Moreover, for some of these microscopes, considerable time is needed to obtain the skills to become a SEM operator. Regarding the scanned area, it is reduced, and the images produced are colorless. Different methods have been developed to preserve the native plant structure. However, sample preparation does not guarantee artifact-free images of some plant tissues (that can suffer shrinkage or other morphological changes) (Neinhuis and Edelmann, 1996; Pathan et al., 2008; Schwab and Hülskamp, 2010; Talbot and White, 2013). Variable pressure SEMs allow direct observation of detached fresh samples. However, samples with a high content of water are still challenging due to, e.g., dehydration. This can be partially solved by using a cool stage in the SEM chamber to “freeze” the sample, though, depending on the tissue, it still can be challenging. Some tissues are extremely delicate and dehydrate very fast.

Another type of microscope often used to characterize plant phenotypes with detail, is the confocal laser scanning microscope (CLSM), which allows the observation of fluorescent signals and produces high-resolution images (Reddy et al., 2007; Reyes-Olalde et al., 2015). It also produces three-dimensional (3D) reconstructions, such as Z-stacks (tridimensional Z-stack images are obtained by merging several two-dimensional images obtained at different focal planes along the volume of a sample). However, it is not appropriate for unstained non-fluorescent structures, the cost of the equipment is rather high, and it is not practical for observing large structures.

Newer technologies such as light sheet fluorescence microscopy are also expanding the limits of phenotypic analysis at the small to medium scale (Reynaud et al., 2015) but also require fluorescence. Tomography has been used to visualize plants and plant organ and tissues, for example using X-rays, as in high resolution X-ray computed tomography (HRXCT), a non-destructive method that allows the extraction of three-dimensional (3D) morphological traits (Dhondt et al., 2010). Another example is optical projection tomography (OPT), a form of tomography combined with optical microscopy, where light and fluorescent markers can be used, and that has also been modified to allow observation of relatively large samples (Lee et al., 2017). Each method can be used for different purposes, such as the observation of the surface or the interior or whole organisms, organs, cells, or subcellular features.

On the other hand, the needs of the manufacturing industry for fast and reliable microscopy, required for rigorous quantitative quality control to guarantee standard sizes and shapes of manufactured objects of different sizes (for example, products manufactured by the automotive, pharmaceutical, electrical, industries, etc.), has led to the evolution of industrial microscopes with practical measurement capabilities. One of these microscopes is an optoelectronic or optodigital 3D color microscope that allows non-destructive observation in non-treated, non-dissected specimens. This microscope employs lenses to magnify an image of an object, as an optical microscope. The magnified image, instead of being directly detected by the human eye, is projected upon a charge couple device (CCD) or complementary metal oxide semiconductor (CMOS) imager that converts incoming photons into electron charges, and images are captured similarly as in a digital camera. These images are displayed on an integrated liquid crystal display (LCD) monitor with high resolution. This kind of microscopes can visualize samples at a long range of magnifications (from 0.1× to 5000×, depending on the lens used). It also allows imaging from different angles, and because the lenses can be removed from the stand, it provides ample freedom with respect of the position of the object to be imaged, which is not common in most microscopes. Moreover, this allows the inspection of larger samples than those visualized in normally used stereomicroscopes.

Other differences with normally used lab stereoscopes include the combination of a motorized stage with high quality of image stitching (the automatic assembly of many single images) that can be used to obtain a single panoramic (large field of view) image. Moreover, it also has a focus stacking function that allows to obtain 2D images that are completely in focus (having a large depth-of-field), by combining different in-focus images along the *Z* axis, in nearly real time.

Moreover, the integrated software and motorized stage allow the reconstruction of 3D full color models without exporting the separate pictures to be processed with other software. To make 3D models, the stage is moved in the *Z* axis by a five-phase stepping motor with a movement range between 49 mm and 0.1 μm. Therefore, it can make reconstructions of a long range of object sizes. 3D reconstructions are made using images captured along in the *Z* axis, and imaging from multiple angles is not required (and in the particular microscope here used, not used to make reconstructions). Panoramic 3D images can also be obtained by using 3D rendering combined with image stitching.

The optoelectronic microscope allows the examination of uneven, rough surfaces without contacting nor damaging them. The software of these microscopes can measure and document parts of different materials and textures, sizes and depths at a precise and semiautomatic pace, and the microscopes themselves are very resistant to heavy duty, intensive use. Moreover, they are user friendly, and do not require special conditions nor extensive maintenance.

However, even when these microscopes have characteristics that appear to suit detailed plant phenotypic analyses, plant science reports that employ them are extremely rare. Until now, we have found a single report, about its use in the field of plant disease in the agricultural context, evaluating fruit skin damage (Klemm et al., 2016). However, it appears that the microscope could perform more diverse analyses in plant samples, in the context of plant development, or mutant characterization, for example. Here, we explored whether these tools, originally designed to meet industrial needs, were indeed also useful and advantageous for the morphological and, in general, phenotypic analyses that are commonly performed by plant development scientists working with different organs and tissues of model plants.

## Materials and Methods

### Plant Material and Conditions

*Nicotiana tabacum* SR1 wild-type plants were grown in soil in pots in the greenhouse. Images of 2- and 3-week-old plantlets are presented in the figures. For *Arabidopsis thaliana*, Columbia (Col), Landsberg *erecta* (L*er*), and Wassilewskija (Ws) wild-type, and *agamous (ag-1)*, *transparent testa 5 (tt5)*, *arabidopsis B-sister (abs)*, *dörnroschen-like 2 (drnl-2)*, and *bolita-D (bol-D)* mutant plants and seeds were used (Yanofsky et al., 1990; Shirley et al., 1995; Nesi et al., 2002; de Folter et al., 2006; Marsch-Martinez et al., 2006; Nag et al., 2007). After seed stratification, plants were grown in soil under greenhouse conditions (375 cm^2^ trays, 15 plants per tray) or in plates on solid half strength Murashige and Skoog (MS) medium, containing 0.8% agar and 0.5% sucrose, in a growth chamber at 22°C with 16/8 h photoperiod for 5 days. The Arabidopsis used for the proof-of-concept (mutant leaf analyses) test were 35 days old (most plants were flowering). Mature seed was used for the color test, and flowers and siliques were obtained from plants older than 7 weeks. Liverworts (*Marchantia polymorpha*) were grown in solid medium (half strength B5 Gamborg medium containing 1% agar and without sucrose) in plates or soil in a growth chamber with the same conditions as Arabidopsis.

### Microscopes

Plant organs were observed and photographed using three different microscopes (stereoscope, electron, and optoelectronic microscopes). The stereoscope was a Stemi 2000-C (Carl Zeiss, Oberkochen, Germany) with a zoom range from 0.65× to 5×, coupled to an Axiocam ERc 5s (Carl Zeiss) with a resolution of 5 megapixels. The electron microscope was a variable pressure electron microscope (VPSEM) EVO 40 microscope (Carl Zeiss). Scanning electron microscopy images were captured using a 20 kV beam, and the signal was collected using the BSD detector. The optoelectronic or optodigital microscope was a VHX-5000 Keyence microscope (Keyence, Osaka, Japan) equipped with a VHX-5100 camera (with a resolution of 18 megapixels) and VHX-Z20R (20× to 200×) and VHX-Z500R (500× to 5000×) lenses. The stereoscope and the electron microscope were used to obtain Arabidopsis *agamous* mutant flower, seedling, and silique images that were qualitatively compared to the images obtained with the optoelectronic microscope. Plant tissue was collected fresh and directly observed with the microscope in these cases. Besides the images indicated for the other microscopes, the optoelectronic microscope was also used to obtain photographs, perform quantitative analyses or reconstruct 3D models of fresh Arabidopsis, tobacco and liverwort organs (or whole seedlings or seed), or fixed Arabidopsis leaves. Subcellular imaging was not performed with any of the microscopes.

### Mutant Phenotype Analyses

Color detection and measurement in Arabidopsis seeds and leaves was performed using the red, green, and blue (RGB) function of the software of the microscope. With this function, the intensity of each RGB color at selected points on the images of seeds or complete leaves was automatically extracted by the microscope-accompanying software. A total of 10 points for mutant and wild-type seed, and 50 points for mutant and wild-type leaves were taken for each genotype.

Mutant leaf analyses were performed in fixed leaves, in order to test whether useful images could also be obtained using fixed tissue. Fixed leaves in a 4% paraformaldehyde solution, of 35-day-old, soil-grown (greenhouse) plants of four genotypes (*bol-D*, Ws, *drnl-2*, and L*er*), were used for phenotypic analyses. Images of their whole adaxial side were acquired at 20× magnification. These images were used to automatically extract quantitative information such as leaf area, perimeter, and maximum and minimum diameter (though the “diameter” variable may be more useful in industrial applications, than in morphological analyses of many plant organs). For this, the semi-automatic area segmentation function of the microscope, using color to recognize the leaves in the images, was used. Images of whole cauline leaves (the first cauline leaf) of 10 *drnl-2* and L*er* plants and rosette leaves of 5 *bol-D* and Ws plants were used for the analyses. Since *bol-D* leaves are curved, they were flattened (pressed against the support surface), and the software segmented area was used to extract the quantitative information.

For epidermal cell counting, a 1000× magnification was used to acquire the micrographs of the adaxial side of the leaves, near their distal tip (two images, one on each side of the main vein, were acquired per leaf). Pavement epidermal cells were manually indicated by clicking on them in the image and semi-automatically counted by the software (using the counting function that keeps track of the number of times the user has clicked in the image).

### Statistical Analysis

Different statistical analyses were performed depending on normality assessment (using a Shapiro–Wilk test). For normally distributed variables, an ANOVA followed by a least significant difference (LSD) test (for the RGB color analysis of Arabidopsis seeds) or Student’s *t*-tests (when comparing only two populations such as in the leaf area and perimeter analyses, and RGB color analyses of leaves) were performed. For the data that did not present a normal distribution, non-parametric tests were performed (Mann–Whitney test for cell number in mutant and wild-type leaves). The tests were performed, and the graphs generated using the SPSS software.

## Results and Discussion

We explored whether an optoelectronic 3D color microscope could be suitable for plant imaging and morphometric analyses, because some of its characteristics (like autofocus, freedom to observe a sample from different angles, and fast acquisition of large depth-of-focus and 3D color images, among others), could be very useful to document plant phenotypes in a practical fashion, but had been barely used for this purpose (Klemm et al., 2016). First, we explored the performance of the microscope using different organs and tissues of some widely studied model plants to test the range of sizes and shapes that the microscope can work with. These tests included the following: (i) imaging plant tissues and cells directly in their growth conditions (soil in pots or medium in plates); (ii) reconstructing direct 3D images of different non-flat, curved, or irregular organs, and as part of this exploration we also qualitatively compared the images obtained with the tested microscope, to those obtained with a stereoscope or SEM commonly used for plant sciences; (iii) reconstructing images of whole organs automatically, from images of sections obtained at high magnification; and (iv) testing the automatic segmentation function, and the extraction of quantitative information regarding shape and color from the images obtained by the optoelectronic microscope. After the general exploration, we performed a leaf size and color, and cell size analysis of loss- and gain-of-function mutants using the microscope as a tool and compared area measurements of leaf cells with ImageJ and the reproducibility of 3D reconstructions using a standard object. Finally, we included a section indicating the challenges faced during the different tests.

### Structure of the Digital Microscope

**FIGURE 1:**
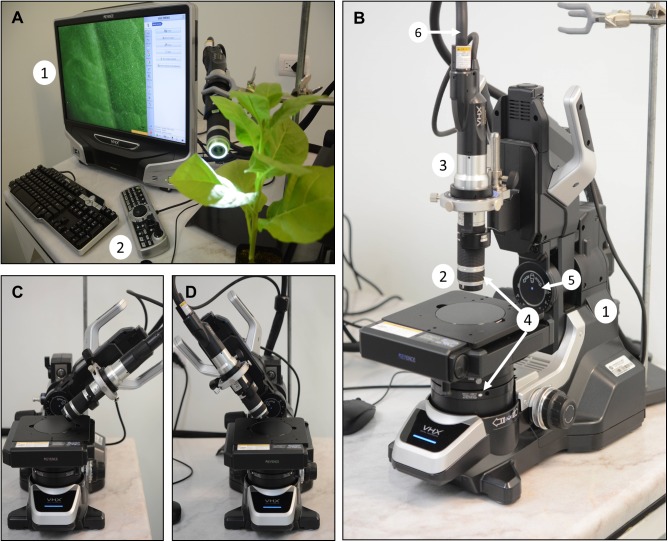
Main components and accessories of the optoelectronic microscope. **(A)** Controller unit, 1: a CPU-LCD screen, 2, console. **(B)** Microscope system unit; 1, free angle observation system; 2, lens (20× to 200×); 3, camera; 4, LED illumination units; 5, wide-angle controller; 6, optical fiber connection. **(C,D)** Examples of observation positions at different angles.

For our tests, we used an optoelectronic Keyence VHX-5000 microscope (**Figure 1**). It can generate high-resolution, large depth-of-field, color images at different magnifications (0.1×–5,000×), depending on the zoom lens used (here, we tested the 20–200× and 500–5000× lenses). The microscope includes the main body (**Figure 1B**), a CPU-LCD screen, a manual controller (**Figure 1A**), and image analysis software as accessories. The main body is composed by four pieces: a motorized stage that allows XYZ movement, zoom lens, camera, and two LED light sources integrated in the body and as a ring in the lens (**Figures 1A,B**).

The lens and camera can be positioned at different angles in the structure (**Figures 1C,D**), and therefore, different sides of an object can be observed without moving it. Furthermore, because the lenses and the camera can be easily disassembled from the main body, there is even more freedom of movement, which appeared to be especially useful for large plants. The lenses could be placed in different holders, in a wide range of configurations. For example, we used a common laboratory clamp stand to hold the lens unit (which includes the camera) at different heights and angles, above, below, or next to the sample facilitating observations of objects and surfaces from different points of view (**Figure 1A**).

### Direct, Non-destructive Observation of Tissues and Organs

First, we explored whether images could be taken directly without detaching tissues from plants or moving them from their original growth conditions, which would be very convenient but is rarely easily achieved with most high-magnification microscopes. For example, directly obtaining detailed images of organs or tissues of large plants growing in trays, pots, or smaller plants growing in plates, is not always easy or even possible using an SEM, many confocal or even optical microscopes. By contrast, the structural configuration of the optoelectronic microscope appeared to be suitable for direct imaging. We tested whether this configuration could be used to directly observe and document plants, and obtained images of a whole young tobacco plant in soil, and also a zoom in the shoot apical meristem region and leaves (**Supplementary Figure S1**). After imaging, the intact plant was brought back to the greenhouse.

Attaching the lens to a clamp stand would also allow the direct observation of the above organs of middle sized plants, as shown in **Figure 1A**. This can allow the visualization of phenotypes at different time points, for a long time during development, from different angles and positions, without detaching the visualized organ or tissue.

**FIGURE 2:**
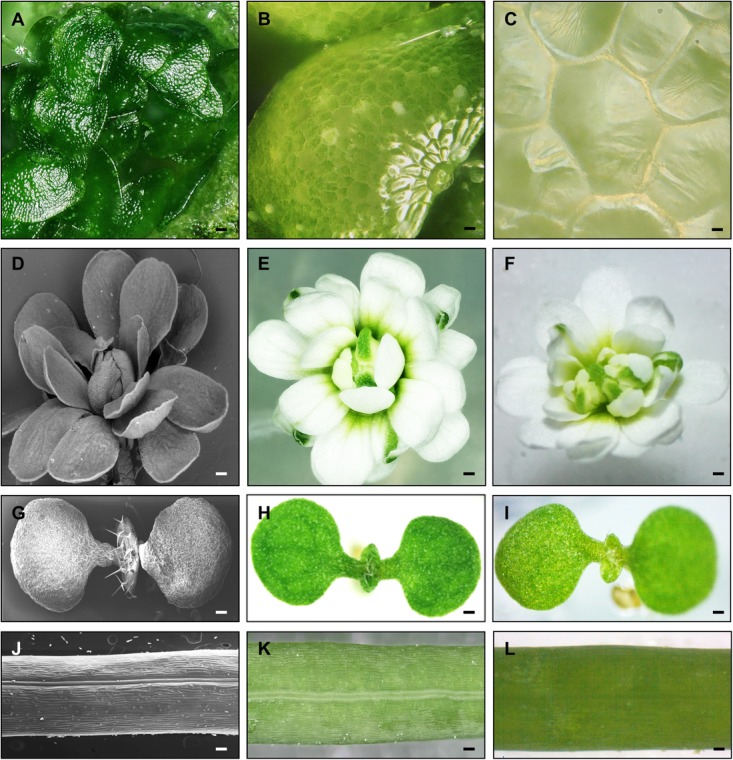
*Marchantia polymorpha* and *Arabidopsis thaliana* organs and tissues. **(A–C)** Images of an *M. polymorpha* gemmae cup, a gemma, and epidermal gemma cells, obtained with the optoelectronic microscope. Images of an *A. thaliana agamous* mutant flower, wild-type seedling, and silique obtained with a scanning electron microscope [SEM **(D,G,J)**]; with the optoelectronic microscope **(E,H,K)** or a stereoscope **(F,I,L)**, respectively. **(A–C)** 200× **(A)**, 500× **(B)**, and 3000× **(C)**. Scale bars: 75 μm **(A)**, 30 μm **(B)**, and 5 μm **(C)**, 200 μm **(D–F)**, 500 μm **(G–I)**, 500 μm **(J–L)**.

Furthermore, the detached lens unit from the main body can be hold in one hand and freely positioned. In this way, images of a liverwort (*M. polymorpha*), a basal plant species emerging as a new model for evolutionary and functional studies (Flores-Sandoval et al., 2015) were obtained. These plants produce non-sexual reproductive structures called gemmae. We used the detached lenses to directly obtain different images of liverwort plants growing in a petri dish, without any further sample preparation. The detached lenses were able to obtain sharp images of a gemmae cup (the structure where gemmae develop and are contained), a gemma, and the epidermal cells of a gemma at magnifications of 200×, 500×, and 3000×, respectively (shown in **Figures 2A–C**). Next, we also tested direct imaging of *A. thaliana* whole seedlings that were growing in medium (which is not possible when using SEM), producing the image shown in **Figure 2H**. All these color images showed a large depth-of-field. The same analysis in an electron microscope would have left these liverwort and Arabidopsis plants non-viable.

During these different observation trials, we found that the tested lenses could autofocus and perform magnification changes in seconds (as long as the changes are within the range of one lens, because it takes minutes to change lenses). Therefore, it appears that this 3D color microscope can be suitable to obtain, in a fast way (when the same lens is used), high-quality images at different magnifications in a non-destructive, real-time fashion, and without any sample preparation requirements.

### Direct Reconstruction of 3D Color Images of Non-flat Plant Organs

In the next test, we evaluated the capacity of the microscope to produce sharp images of non-flat surfaces. When the surface of an object is not flat, or irregular, it is challenging to display the whole object in focus in a single image. The optoelectronic microscope tested can make quick automatic scans through the focal range of samples in observation, recognizing areas of focus at different distances from the sample, and use the acquired images to digitally reconstruct a fully focused color image in a short time. A first example of this reconstruction function, is given in one of the above described experiments. The curved shape of the liverwort gemmae posed an interesting challenge to obtain focused images of the whole surface. For the image of the gemmae, we used the reconstruction function, and could obtain a sharp image of the complete surface of the curved structure, as presented in **Figure 2A**.

We further tested this ability of the optoelectronic microscope by imaging different plant tissues of Arabidopsis and comparing (in a qualitative fashion) the resulting images to those obtained with other types of microscopes (SEM and a stereoscope). First, we obtained captures of flowers of the *agamous* mutant, where the development of reproductive organs and meristem determinacy are impaired. These developmental alterations result in conspicuous mutant flowers that are characterized by the presence of supernumerary sepals and petals (Yanofsky et al., 1990). Using the optoelectronic microscope, we set the range of planes from the middle to the top of the mutant flower that would allow the full visualization of the flower from the top, and the microscope acquired the images in the different focal planes at a fast speed, resulting in an entirely in-focus final color image (**Figure 2E**). Furthermore, the microscope also automatically acquired information for each pixel in each plane. Using the images of the different planes, the software of the microscope compiled Z-stacks to produce a 3D model where details of the mutant flower could be appreciated even better. The 3D model generated for the *agamous* mutant flower (presented in **Supplementary Figure S2A**) also contained useful extractable information on depth and height of the different parts of the flower, which in principle could be used to make statistical analyses. Depending on the 3D model, some models can be turned in different directions, allowing the user to visualize and measure distances from different sides of the sample. We also obtained images of the surfaces of *in-vitro* grown 1-week-old *A. thaliana* seedlings (**Figure 2H**), and specific regions of developing siliques (**Figure 2K**). These reconstructions of different captures allowed the visualization of all planes in focus (**Figures 2E,H,K**). We qualitatively compared the images to those obtained using a SEM and a stereoscope. The obtained SEM images are sharp, with high resolution, and with a large depth-of-field (**Figures 2D,G,J**), but in gray scale. Stereoscope (equipped with a color CCD camera) micrographies are in color, but lack sharpness in some planes (**Figures 2F,I,L**), depending on the depth of an object, and post-processing would be required to obtain images where all planes are in focus. By contrast, the 3D microscope could directly obtain full-color images that were focused in the different planes of the flower, silique, or seedling. The resolution may be less than in images obtained by the SEM, but samples remain intact (or viable) and can be measured repeatedly.

### Image Reconstruction of Whole Organs at High Magnification

**FIGURE 3:**
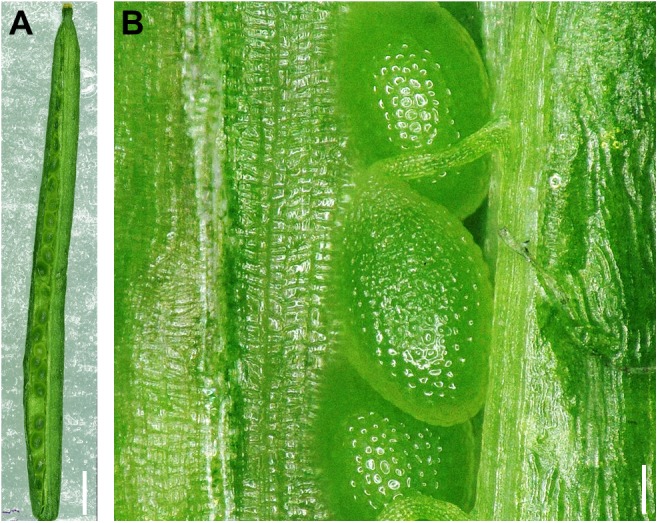
Image reconstruction of a whole Arabidopsis silique at high resolution. **(A)** Reconstruction of a whole wild-type Arabidopsis silique using images taken at 200× (Col). **(B)** Example of a magnified region of a reconstructed image of a whole silique. Scale bar in **(A)**: 1 mm and **(B)**: 100 μm.

We next tested the ability of the microscope to scan a relative large area and compile different sections in a single image. Sometimes, when analyzing plant tissues or organs, and depending on the magnification used to visualize their surface, it can be difficult to precisely define where the observed area was localized in the whole organ (for example, a small region in an Arabidopsis silique). Other times, detailed information of whole organs is required to document phenotypes that can be carefully analyzed afterward. Using a motorized stage and automatic image assembly, the optoelectronic microscope can reconstruct images of large areas with high magnification and high resolution in a short time. We tested its performance and suitability for plant organs by imaging, at a magnification of 200×, contiguous regions along whole siliques of the model plant *A. thaliana* and then using all images to reconstruct the complete surface of one side of a silique in a single image. **Figure 3A** shows one of the reconstructed images. Any point along a reconstructed whole silique image can be zoomed in and visualized with high resolution (**Figure 3B**). This kind of images allows the documentation and detailed analysis of phenotypes of whole “medium-sized” plant organs (in this case around 1 cm), where small areas at any position along the organ can be also visualized. It is also possible to obtain these images in other light microscopes. However, in many cases, images must be taken manually, and they need to be processed with secondary software to obtain the reconstruction. Here, the time to obtain the reconstructed image of the whole silique at 200×, without sample preparation, was short (around 1.5 min including time to obtain 20 images and perform the reconstruction that represents an area of 1.3 cm^2^). This is very convenient because it would allow working with larger numbers of samples than with common lab microscopes. When phenotyping a large number of mutants or plants grown in different conditions, or obtaining morphological data that will be subjected to statistical analyses, this can be an advantage.

### Automatic Morphometric Analyses of Plant Organs and Tissues

In the next test, we explored the semi-automatic segmentation, line, perimeter, area, and height measurement; and color quantification functions of the microscope. As with other tests, we used different organs and tissues of model plants to know whether these functions could be used in samples of the different sizes and shapes commonly studied in plant sciences.

**FIGURE 4:**
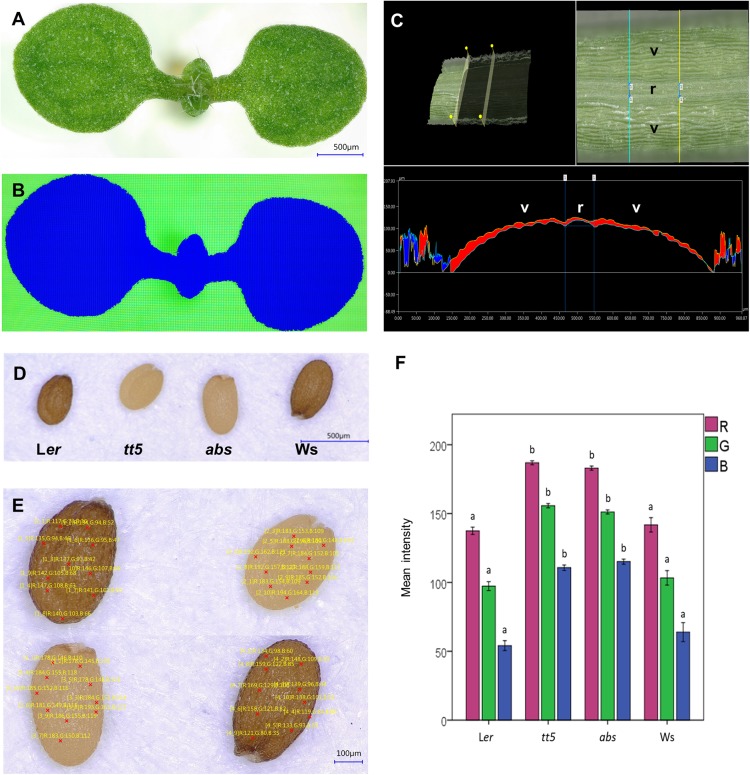
Morphometric and color analyses of plant tissues. **(A,B)** Semi-automatic area segmentation of the aerial organs of an Arabidopsis seedling, depth profile of a silique, and seed color analysis. Original seedling image **(A)**, seedling area segmented by the software **(B)**. **(C)** Example of a depth profile extracted from a 3D image of a region of a silique. **(D)** Reconstructed images of dry wild-type and mutant seeds that have different colors, obtained from individual captures of seed regions at 200×. **(E)** Ten random points were used to analyze the RGB color composition of the four different seeds. **(F)** Bar graph of the RGB color composition data extracted from the images of wild-type and mutant seed. Different letters indicate statistical significances between groups; L*er* vs. *tt5* and Ws vs. *abs* **(E)**. Valve (v); replum (r).

Morphometric studies measure and compare quantitative differences in the physical form of a plant, organ, tissue, or cell. Optodigital microscopes can perform automatic, direct measurements of distance, area, and depth of manufactured objects in a live fashion. Calibration data are integrated into the digital microscope, so it can be used without calibration. However, to obtain higher precision, a calibration scale can also be used. The included software is intuitive, so users can perform reproducible and reliable measurements and quantifications with little training. Some of these measurements include the distance between two points, radius of specified circumferences, distance between parallel lines, length of perpendicular lines, and angles. Also, the software can automatically segment an area within the acquired image, using differences in contrast, color, or manually defined characteristics to define boundaries. To test the color-based area segmentation function, we used *A. thaliana* seedlings. An image of a 2-week-old wild-type seedling was obtained (**Figure 4A**), and the area corresponding to the seedling (green in the image) was automatically detected using the microscope software (**Figure 4B**). Once the area was delimited (indicated as the blue area in **Figure 4B**), the software was used to automatically extract and export quantitative data, such as perimeter, area, and diameters as a .csv (comma separated values) file, from which the data were taken and are presented in **Supplementary Table S1**. The area segmentation function was also used to recognize the area of a senescent Col wild-type rosette leaf, where the intensity of the color was not uniform (**Supplementary Figure S3**). The color of different points was used for segmentation, and the area was successfully identified.

Next, we tested the live linear measurement function of the microscope using as an example the epidermal cells of a *Marchantia* gemma. An example of cell length measurements is presented in **Supplementary Figure S2B**. In the measurements presented in this example, the curvature of cells is not taken into account. We also explored 3D plant surface characteristics, using 3D reconstructions from the images captured at different planes. From these reconstructions, valuable information such as the height of objects and perimeter measurement of a curved surface, can be extracted. We explored this function by imaging a section of an *A. thaliana* fruit. The Arabidopsis fruit is externally composed by two concave valves, which protect the developing seed inside them, and are bound to each other on both sides. In between the region where the valves contact each other, there is a region named replum, and the cell files next to it are called “valve margin.” These regions are very important because they allow the fruit to open when the seed is ripe, dispersing the mature seed. Quantitative evaluations of the characteristics of these tissues are useful to understand, for example, gene function during their development. However, sometimes quantification is difficult in these non-flat structures. **Figure 4C** presents a 3D reconstructed segment of a silique and a virtual cross section where distances between and size of specific regions such as valves and replum were measured and are indicated. This information is very valuable when studying the effect of mutations in certain genes in the formation of these tissues.

**FIGURE 5:**
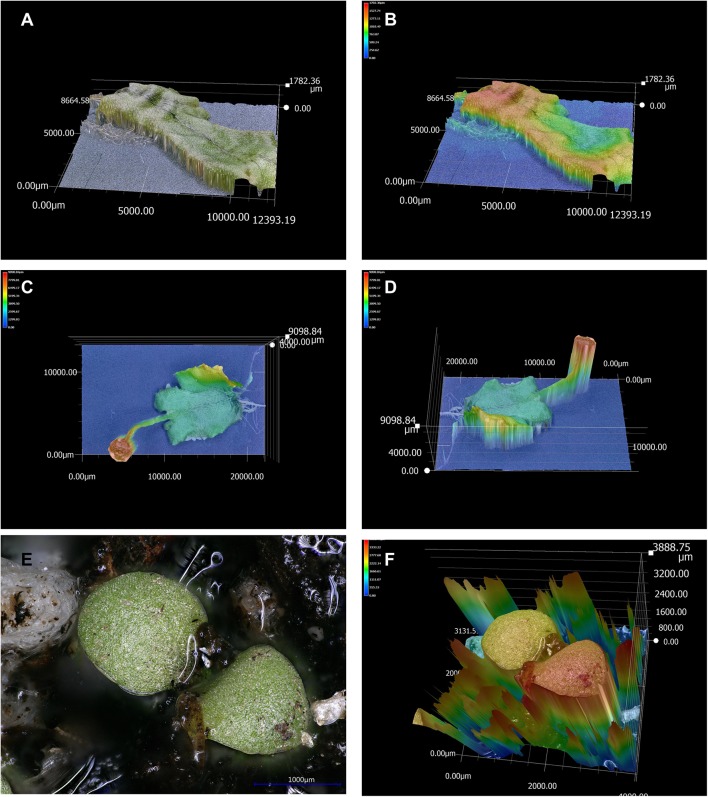
3D model and topographic maps of liverwort and tobacco plants. **(A)** 3D reconstruction of a liverwort thallus without background reflection. **(B)** Topographic representation (colors indicate different heights) of the liverwort thallus. **(C)** Topographic representation of a liverwort thallus with an antheridiophore (male reproductive structure) in the *X* and *Y* axes. **(D)** Topographic profile of the same thallus as in **(C)**, in *X*, *Y*, and *Z* axes. **(E)** Micrograph of a tobacco seedling in soil, presenting light reflection. **(F)** Topographic profile of the 3D reconstruction of the tobacco seedling in **(E)** exemplifying how the reflected lights affects depth calculation.

Another example of a 3D reconstruction, in **Figures 5A,B**, shows the reconstruction of a whole organ, a liverwort thallus as a 3D model, and topographic map, where the height of each pixel is indicated by a color scale. **Figure 5C** shows the topographic map of another whole liverwort thallus that includes an antheridiophore, the male reproductive structure.

We also explored whether the topographic function could be used to image a whole *A. thaliana* seed, which has a round shape, and present a virtual cross section and the topographic map in **Supplementary Figures S2C,D**.

The reconstruction of middle-sized models was relatively fast. The approximate time to complete the reconstruction of an area of 2.5 cm^2^ at a magnification of 20× and an approximate height of 1.2 cm is around 30 s. A reconstruction of an object of the same area and height at a magnification of 200× takes place in about 5 min, including imaging and model calculation.

### Direct Color Quantification (RGB)

The software can also extract and export color information from an image. Color analysis has become of great importance in the plant field in the last years. It allows the identification of variations in the accumulation of different pigments, which can aid the comparison of mutant phenotypes, taxonomic variations, and plant disease diagnostics, among others.

Data on the RGB intensity values of selected points in the color images produced by the microscope can be exported by means of a csv file for further processing.

To test whether this function could be useful for color analysis in plant samples, we performed large area reconstructions of *A. thaliana* seeds of different genotypes known for their color diversity (presented in **Figures 4D,E**). For the color test, we included wild-type and mutant seeds characterized by a reduced content of pigments (**Figure 4D**). The wild-type reference accessions were Landsberg *erecta* (L*er*) and Wassilewskija (Ws) that have brown seeds. For the mutants, we included a loss-of-function allele of *ARABIDOPSIS B SISTER* (*ABS*; also called *TRANSPARENT TESTA 16*), a MADS domain transcription factor. This gene is involved in endothelium development, a tissue containing proanthocyanidins that upon oxidation provide the brown color to the testa of the seeds. Therefore, in the *abs* mutant, where this tissue is missing, the seeds are yellow (Yanofsky et al., 1990; Shirley et al., 1995). The other mutant genotype was *transparent testa 5* (*tt5*). This mutant is deficient in the enzyme chalcone isomerase that participates in the flavonoid pathway that produces proanthocyanidins. The mutation hinders pigment biosynthesis, causing that the produced seeds are also yellowish instead of brown (Nesi et al., 2002). We used the microscope software to evaluate the RGB composition in 10 randomly selected points of each different seed image (**Figure 4E**). The data were then exported to as a .csv file (**Supplementary Datasheet S1**). The exported values were used to generate a barplot that illustrates the color differences of the seeds as RGB values (**Figure 4F**), allowing the quantitative evaluation and statistical analysis of the color alterations of the mutant seeds. In the examples of this section, the data was obtained in minutes, without the need of separate software for image analysis, illustrating the practicality of 3D color microscopes and their software for efficient analysis of areas and perimeters of flat and curved tissues, and altered color phenotypes.

### Test: Quantitative Analysis of Leaf Phenotypes of Arabidopsis Gain- and Loss-of-Function Mutants

After the different tests, we used the 3D color microscope to gain more detailed information about the function of the Arabidopsis *BOLITA/DÖRNROSCHEN-LIKE/ENHANCER OF SHOOT REGENERATION 2/SUPPRESSOR OF PHYTOCHROME 2 (BOL/DRNL/ESR2/SOB2)* gene. This gene regulates organ development. Phenotypes in the embryo, cotyledons, general plant size and flower organs of gain- and loss-of-function mutants, and regenerating roots in overexpression lines have been extensively studied (Ikeda et al., 2006; Marsch-Martinez et al., 2006; Ward et al., 2006; Chandler et al., 2007; Nag et al., 2007; Chandler and Werr, 2017; Durán-Medina et al., 2017). However, we noticed previously uncharacterized phenotypes, particularly in the cauline leaves of the *drnl-2* loss-of-function allele. Therefore, we used the 3D color optoelectronic microscope to further characterize this phenotype and other characteristics. In this analysis, the *bol-D* gain-of-function mutant allele was also included.

While previous analyses were done using fresh tissues, the analysis of mutant leaves was done using fixed leaves, to test whether the images were good enough to perform measurements, considering that in some experiments, many leaves have to be analyzed at the same time, and fixing them could be an option.

*Drnl-2* and L*er* plants were already flowering. Their cauline leaves seemed to be different in size, and therefore, their first cauline leaves were analyzed. However, for the *bol-D* and Ws leaves, because of the *bol-D* phenotype (a very compact plant where the stem does not elongate normally, and leaves lack a petiole), it is hard to distinguish between cauline and rosette leaves, and leaf number of the latter. Since cauline leaves are normally at the upper part of the plant, leaves at the middle level were used, and compared to middle rosette leaves of the wild-type plants. The distal part of leaves was chosen because cell division decreases, and cell elongation starts earlier at the tip of the leaf than at its base.

**FIGURE 6:**
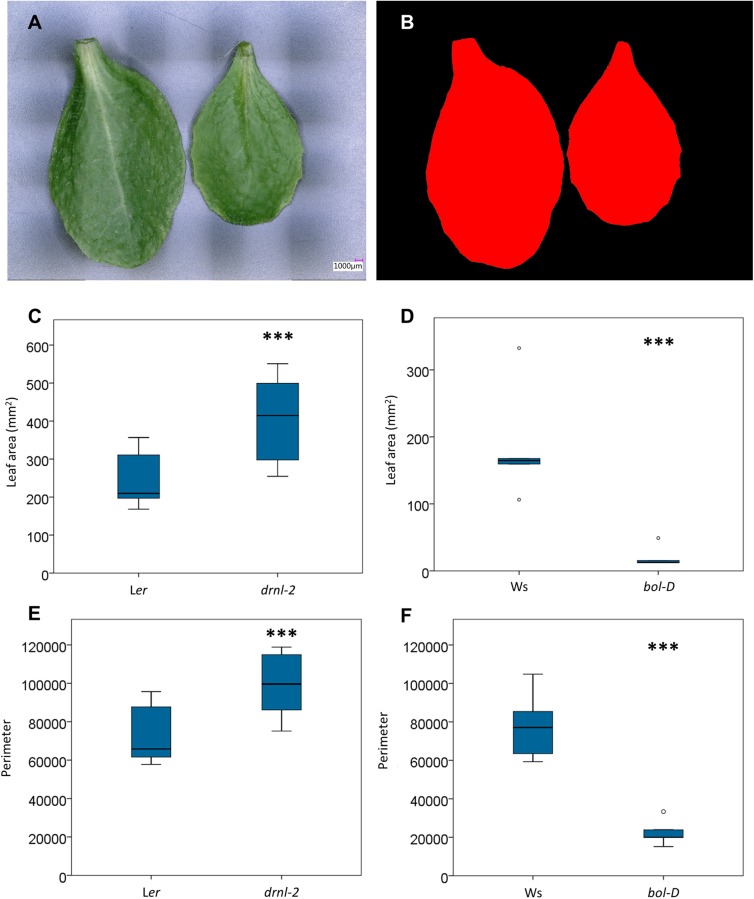
Area and perimeter of loss- and gain-of-function mutant and wild-type leaves. Measurements were automatically extracted from images of whole cauline and rosette leaves. **(A,B)** Example of semi-automatic area segmentation **(B)** from an image of wild-type L*er* cauline leaves **(A)**. **(C,D)** Box plots of the area *drnl-2* and L*er* cauline leaves **(C)** and *bol-D* and Ws rosette leaves **(D)**. **(E,F)** Box plot of the perimeter (micrometers) of *drnl-2* and L*er* cauline leaves **(C)** and *bol-D* and Ws rosette leaves. To evaluate whether there were statistical differences in area or perimeter, a Student’s *t*-test was performed. Mutant and wild-type leaves were different (*P*-value <0.001, represented by three asterisks). *N* = 10 *drnl*-2 and L*er* cauline, and 5 *bol*-D and Ws rosette leaves.

First, images of whole cauline leaves of 10 of the loss-of-function mutant (*drnl-2*) and L*er* plants were obtained (**Figure 6A**). Then, employing the area segmentation function (**Figure 6B**) of the software, area, perimeter, and maximum and minimum diameter measurements were automatically extracted (**Supplementary Table S2**, diameter may be more relevant in industry). The same was done for rosette leaves of five gain-of–function mutant (*bol-D*) and five Ws plants. Interestingly, the cauline leaves of the loss-of-function mutant presented a larger whole leaf area than L*er* (**Figure 6C**). On the other hand, as previously reported (Marsch-Martinez et al., 2006), the leaves of the gain-of-function allele (*bol-D)* presented a smaller area than Ws (**Figure 5D**). The same occurred with the leaf perimeter (larger in the loss-of-function and smaller in the gain-of-function allele, each with respect to their own wild-type ecotypes, **Figures 6E,F**).

**FIGURE 7:**
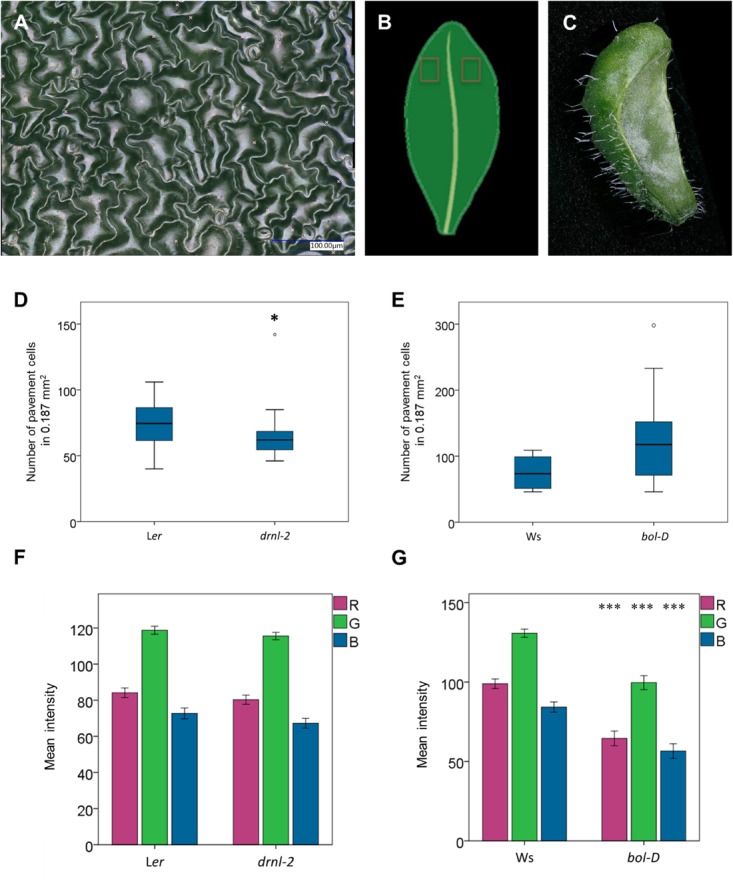
Leaf epidermal cell and color analyses. **(A)** Example of an image of epidermal cells of a *drnl-2* leaf, used to visualize and count pavement cell number (indicated with a light x). **(B)** Scheme of the leaf region scanned to obtain the epidermal images. **(C)** 3D reconstruction of a whole *bol-D* leaf. **(D,E)** Box plots comparing the number of pavement cells in the scanned area. For statistical analyses, a Mann–Whitney test was performed (*P*-values <0.05 were considered significant), *N* = 20 images for *drnl-2* and L*er*, 16 images for *bol-D*, and 10 images for Ws leaves. **(F,G)** Mean value of the intensities of red, green, or blue (R, G, B, respectively) colors at 10 (*drnl-2* and L*er*) or 5 (*bol-D* or Ws) points per leaf image. To evaluate whether there were statistical differences in color intensities, a Student’s *t*-test was performed. There was no statistically significant difference between *drnl-2* and L*er* leaves, but there was between *bol-D* and Ws (*P*-value <0.001). *N* = 10 *drnl*-2 and L*er* cauline, and 5 *bol-D* and Ws rosette leaves.

Second, we used the higher magnification lens to obtain images of the epidermal cells of these leaves (**Figure 7A**). Images at both sides of the main vein, at the distal regions on the adaxial side of the leaves were obtained (**Figure 7B**). These images were then used to manually mark the pavement cells in them, using the software, and then, employing its automatic count function, obtain the total number in a determined, comparable area (187,334 μm^2^). The number of pavement cells in that area was then statistically analyzed. Some variability was found in these leaves, but *drnl-2* leaves appeared to have, in general, fewer cells per analyzed area than wild-type leaves (**Figure 7D**). On the other hand, *bol-D* leaves appeared to have a larger number of pavement cells in the same analyzed area that wild-type leaves, though this was not statistically significant (**Figure 7E**), possibly due to the larger variation observed in these samples and the difficulty to identify leaf number at the age at which plants were analyzed.

Next, the microscope was also used to document the peculiar phenotype of *bol-D* leaves. These leaves are not flat, and therefore, obtaining images where this peculiarity can be fully appreciated in a normal stereoscope or microscope is challenging. However, with the optoelectronic microscope, 3D models where the curvature of these leaves can be observed were obtained, as presented in **Figure 7C**.

Finally, since the color of *bol-D* leaves seemed to be different (darker) to the color of wild-type leaves, we used the microscope to quantify and evaluate this difference. We also included *drnl-2* leaves and compared them to their respective wild-type leaves. To make the comparison, color intensity in the RGB color system of 10 selected points was automatically extracted from each *bol-D* and L*er* whole leaf image. For the *drnl-2* mutant and wild-type leaves, color information was extracted for five points of each whole leaf image. The extracted red (R), green (G), and blue (B) information was then subjected to statistical analyses that indicated that the intensities of the three colors were different between Ws and *bol-D* (**Figure 7G**). The RGB color model is an additive model where any color can be obtained from the addition of different intensities of the three colors. Zero intensity of the three colors represents black, and the highest intensity of all three represents white. The intensities for RGB were all lower in *bol-D*, which correlates well with the darker color observed at simple sight. On the other hand, no statistical differences were detected for *drnl-2* and its wild type (**Figure 7F**).

In conclusion, some phenotypic aspects of Arabidopsis loss- and gain-of-function mutant leaves were documented and quantified at different magnifications. With the same microscope, images of both whole leaves and epidermal cells were obtained, and used to directly extract quantitative data (area, perimeter, diameters, cell number, and color intensity). Moreover, 3D images from the curved leaves were generated automatically. The microscope was a practical and efficient tool to characterize the different studied aspects of the mutant phenotypes.

### Comparison and Reproducibility of Measurements

To explore and illustrate the precision of measurements made using the software of the microscope, at least in images obtained at low and intermediate magnification, area reconstructions of a centimeter in a Vernier scale at 20× and 100× magnification were made. **Supplementary Figure S4** shows these reconstructions with the scale bar generated by the software of the microscope corresponding to the 5000 μm of the Vernier scale, matching the correct distance.

The images obtained with the microscope can be exported as .jpeg or .tiff files with 16 bit resolution (with RGB data from each pixel). This allows post-imaging analyses using different software (as done in Massange-Sánchez et al., 2016). Therefore, alternative workflows are possible, for example, if a very large number of plants should be analyzed, or different analyses not available in the microscope software are required. For example, live imaging of epidermal cells, potentially over time for growing leaves, for multiple plants can be done, images can be exported, and batch processing of images using alternative (open source) software tools can be performed.

**FIGURE 8:**
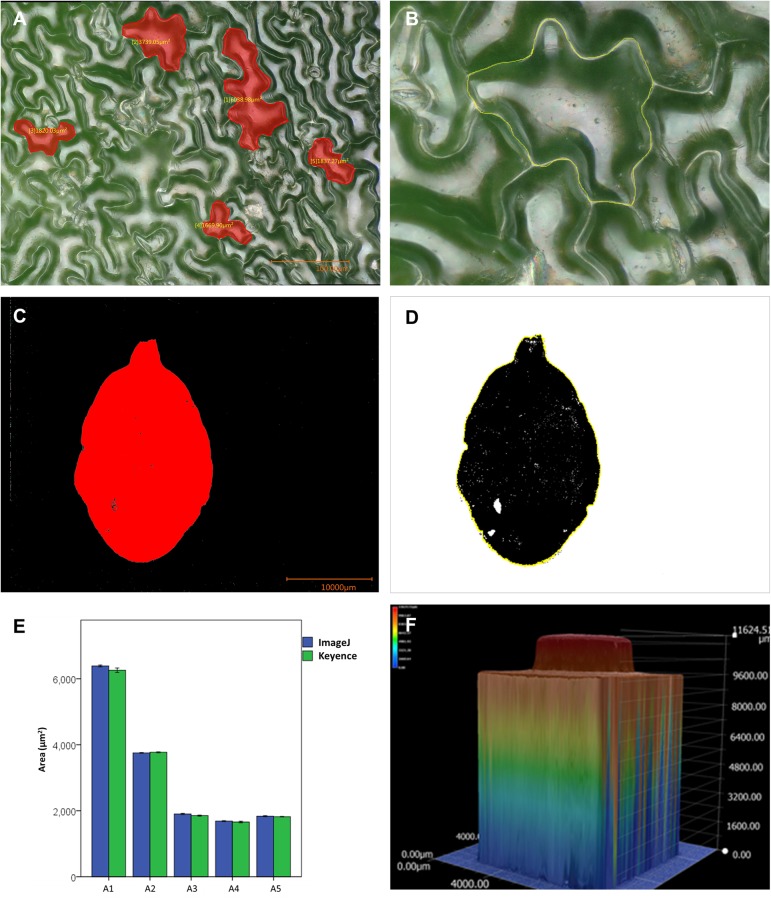
Comparison of cell and leaf area measurements, and topographic representation of a standard object. **(A)** Micrograph of the epidermis of a wild-type Ws Arabidopsis rosette leaf, the selected cells are shown in red. **(B)** A manually selected cell of Arabidopsis using ImageJ, the marked contour is shown in yellow. **(C,D)** Semi-automatic segmentation of the area of an Arabidopsis leaf with the microscope software of the microscope **(C)**, and with ImageJ **(D)**. **(E)** Graph depicting cell (cells A1 to A5) area measurements performed with the microscope software and ImageJ, five measurements per cell with each software. **(F)** Topographic representation of a 1 × 1 Lego piece, the color scale indicates height.

To illustrate this, an image of one of the Ws wild-type rosette leaves was exported and analyzed employing a commonly used image analysis software, ImageJ (Schneider et al., 2012, version 1.49). Repeated measurements (five measurements) of five cells in the image (**Figure 8A**) were performed using both the microscope software and ImageJ. The cells were manually delineated in both cases (**Figure 8B**). **Figure 8E** illustrates the results of the measurements for each cell (measurements can be found in **Supplementary Table S3**). Though variation was observed, statistical tests indicated that measured cell areas were the same. A part of the variation observed could be due to differences in the manual delineation of the cell by the user.

The semi-automatic segmentation function was also used to recognize a leaf in the image presented in **Supplementary Figure S5A**. Color-based area segmentation using the semi-automatic function available in the software of the microscope allowed the correct recognition of the leaf area (**Figure 8C**). This function can also be used in live images and illustrates that the microscope software can segment an area in a direct and simple fashion. The area of the leaf was also correctly segmented by ImageJ when the thresholding function with hue filters was used (**Figure 8D**), and both programs measured roughly the same area (291 mm^2^).

Finally, to test their reproducibility, 3D reconstructions of a common standard object (a 1 × 1 Lego block) were made. To reduce light reflection, the piece was painted with matt paint, which allowed better imaging and reconstruction (**Supplementary Figures S5B,C**). A topographic representation is presented in **Figure 8F**. Five independent reconstructions were made and their height was measured. The measurements were very similar in the independent reconstructions and are presented in **Supplementary Table S4**. 2D images were also used to measure width and length. We concluded that measurements performed with the microscope are reproducible and the proportions are well kept. The obtained information from a depth composition analysis may be accessed as images and also through statistic reports that provide information about cross section profiles of the 3D model. Images and statistic reports may be exported *via* USB, or the microscope can be connected *via* Ethernet to a network to have remote access through another computer to all the files. This allows further analyses using other software.

### Light Reflection Can Obstruct Image Reconstruction and Area Segmentation

In general, the performance of the microscope was very good when imaging the organs and tissues described above. However, we found that there is still room for improvement in imaging conditions and software capabilities, and we describe below some of the challenges identified during this work.

The main problem we encountered was caused by reflected light in some samples, which hindered both area segmentation, and image and 3D model reconstruction. An example of this can be seen in **Figure 7A**. There, light reflection in the leaf surface hindered the semi-automatic segmentation of cells. The image, however, was good enough to allow the user to distinguish individual cells and manually outline them (**Figures 8A,B**). When the user needs to know the number of cells, counting can be done semi-automatically (using the counting function that adds a number each time the user clicks in the image). The problem of light reflection affecting cell segmentation was found in images obtained both in fixed (**Figures 7A**,**8A,B**) and fresh leaves (**Supplementary Figure S1C**). Semi-automatic segmentation of cells would allow faster analyses (such as cell area and number, among others), and modifying light sources may help to diminish the reflected light affecting it.

Light reflection can also affect 3D models and topographic maps. An example is shown in **Figures 5E,F**, where a whole tobacco seedling is presented. Water in the soil reflected light (**Figure 5E**) and this affected the depth calculation, which resulted in an unsuccessful reconstruction of the topographic profile (**Figure 5F**). Another example is illustrated by the attempts to image and make 3D reconstructions of a 2 × 2 Lego piece. The original piece presented high light reflection, and this affected 3D reconstructions and topographic representations, as shown in **Supplementary Figures S5D,E**. In order to image and make better 3D reconstruction of Lego pieces, they were painted using matt paint (as in the images shown in **Figure 8F** and **Supplementary Figures S5B,C**).

The reflected light can also affect reconstructions at higher magnifications, as can be observed in the reconstruction of a 3D model and topographic profile of a region of the epidermis of an Arabidopsis Col wild-type cauline leaf where high reflection is present (**Supplementary Figures S6A,B**). A trichome is present but it cannot be recognized well in the image due to the surrounding reflection. With less reflected light, the reconstructions and profiles improve. An example of a partial reconstruction and topographic profile of a region of the epidermis of the same Arabidopsis leaf, where a part of a trichome can be visualized, is presented in **Supplementary Figures S6C,D**.

We also tried to obtain 3D images of Arabidopsis roots growing in plates, or GUS stained roots, with the microscope. In these cases, the images obtained included reflected light that reduced the quality of the images or hindered the proper semi-automatic segmentation of cells. Therefore, some phenotypic analysis may require further adjustments to the illumination sources, or sample processing protocols to allow better image acquisition.

There are other general points that should be taken into account when using this microscope, or that could be improved. One of them is that 3D reconstructions are performed in the *Z* axis and depend on lens position, which can be turned from -30° to +90°, but the reconstructed object will present background equivalent to its height at the angle at which it was imaged. The possibility of making 3D reconstructions using images from different angles would be very useful. Moreover, it would be very useful if the software could export the *Z* axis (depth) information for each pixel in the image (and even better, each pixel in the segmented area) together with its *X*- and *Y* coordinates.

A second point is to be considered is that if very precise measurements are required, the producer recommends performing manual calibration of the microscope, though this is not needed for general analyses. However, care should be taken when measuring 2D images obtained by focus stacking. To obtain precise measurements it is better to focus on the plane to be measured. When performing live measurements, the plane to be measured can be first focused at a high magnification, and then measured at a lower magnification.

Another improvement in morphometric analyses would be the possibility to take curvature into account when performing linear and area measurements.

Finally, another aspect that could be improved in the software would be color analysis. The possibility to subtract and present summary statistics for the three color channels for an image or a region of an image (for example, a whole organ, or a cell, segmented from the background, instead of information for specific selected points) would allow more complete analyses. Moreover, while area segmentation based on brightness can be performed automatically, it becomes semi-automatic when it is based on color, because the user needs to manually set the thresholds for segmentation.

Nevertheless, we consider that optoelectronic 3D color microscopes can be very useful and practical tools in plant phenotypic characterization of many plant organs and tissues.

## Conclusion

The growing interest on documenting plant, plant organ, and tissue characteristics for multiple aims is a continuous driving force in the search of faster, cheaper, or just practical observation and analysis tools in plant science. Optoelectronic, 3D color microscopes are used in different industrial fields, though not so much in the field of life sciences, and barely plant sciences (Klemm et al., 2016). Here we explored the capabilities and potential of a 3D color microscope as a user friendly, reasonably fast, versatile, and moderate cost tool for plant phenotypic and morphometric analyses. In our tests, the microscope could produce high-resolution, large depth-of-field color images of many of the organs tested with improved focus range from small to large areas. It also allowed easy documentation of image metadata and quantitative information (linear, surface, depth, and color), increasing the possibilities for both image and *in vivo* measurements, providing a practical instrument to study plant form directly and time-efficiently. Furthermore, neither special room conditions nor extensive training are required. There are still some challenges to further improve its performance with specific tissues, but we consider that it is worth for the plant science community to be aware, and to further explore and exploit the potential of 3D color microscopes.

## Author Contributions

HL-R, AG-F, YD-M, SF, and NM-M conceived and the designed the experiments. HL-R, AG-F, YD-M, DD-R, and LS-S performed experiments or analyzed data. HL-R, AG-F, DD-R, and YD-M prepared the figures. HL-R, AG-F, SdF, YD-M, and NM-M prepared the manuscript. NM-M edited the last version. All authors read and approved the final manuscript.

## Conflict of Interest Statement

The authors declare that the research was conducted in the absence of any commercial or financial relationships that could be construed as a potential conflict of interest.
