# Supplementary material for: Non-destructive Plant Morphometric and Color Analyses Using an Optoelectronic 3D Color Microscope
Source: Front Plant Sci. 2018 Sep 25;9:1409. doi: 10.3389/fpls.2018.01409 (PMC6167917; doi:10.3389/fpls.2018.01409)
Supplement: Supplementary file 1 [file Table_1.DOCX]

| **Title** | **Data of seedling maximum area** | | | | | | | |
| --- | --- | --- | --- | --- | --- | --- | --- | --- |
| **Date** | 27/07/2017 10:28 a.m. | | | | | | | |
| **Comment** |  | | | | | | | |
|  | Area | Unit | Perimeter | Unit | Diameter max | Unit | Diameter min | Unit |
| **Average** | 3549715 | µm² | 16214 | µm² | 3922 | µm | 1431 | µm |
| **Standard**  **deviation** | 0 | µm² | 0 | µm² | 0 | µm | 0 | µm |
| **Max** | 3549715 | µm² | 16214 | µm² | 3922 | µm | 1431 | µm |
| **Min** | 3549715 | µm² | 16214 | µm² | 3922 | µm | 1431 | µm |
| **Total** | 3549715 | µm² | 16214 | µm² | 3922 | µm | 1431 | µm |
| **Principal** |  | | | | | | | |
| **No.** | Area | Unit | Perimeter | Unit | Diameter max | Unit | Diameter min | Unit |
| **1** | 3549715 | µm² | 16214 | µm | 3922 | µm | 1431 | µm |
|  | Area | Unit |  |  |  |  |  |  |
| **Total area** | 3549715 | µm² |  |  |  |  |  |  |
| **Count** | 1 | pzs |  |  |  |  |  |  |
| **Area relation** | 24 | % |  |  |  |  |  |  |
| **Total area of region** | 14806285 | µm² |  |  |  |  |  |  |

**Supplementary Table S1. Example of extracted data from an image of wild type Arabidopsis seedling.**
