# Supplementary material for: Non-destructive Plant Morphometric and Color Analyses Using an Optoelectronic 3D Color Microscope
Source: Front Plant Sci. 2018 Sep 25;9:1409. doi: 10.3389/fpls.2018.01409 (PMC6167917; doi:10.3389/fpls.2018.01409)
Supplement: Supplementary file 3 [file Table_3.DOCX]

**Supplementary Table S3.** **Area measurements of 5 epidermal pavement cells of an Arabidopsis rosette wild type leaf**. Each cell was manually delineated and measured 5 times using the microscope software or ImageJ, and the measurements were compared and statistically analyzed by a Student´s T test. None of them showed statistically significant differences. The area is indicated in μm^2^. SD Standard deviation. CV Coefficient of variation.

| Cell: | Stats. | **Keyence** | **ImageJ** | **p-value** |
| --- | --- | --- | --- | --- |
| Cell 1 | Average | 6257.34 | 6387.68 | 0.109635 |
|  | SD | 149.206 | 62.9894 |  |
|  | CV | 2.38% | 0.99% |  |
| Cell 2 | Average | 3772.76 | 3752.55 | 0.314099 |
|  | SD | 33.9804 | 24.8176 |  |
|  | CV | 0.90% | 0.66% |  |
| Cell 3 | Average | 1852.01 | 1900.69 | 0.0866178 |
|  | SD | 35.6011 | 42.889 |  |
|  | CV | 1.92% | 2.26% |  |
| Cell 4 | Average | 1658.65 | 1685.64 | 0.380316 |
|  | SD | 55.3602 | 34.0544 |  |
|  | CV | 3.34% | 2.02% |  |
| Cell 5 | Average | 1819.45 | 1835 | 0.347764 |
|  | SD | 16.3771 | 30.7785 |  |
|  | CV | 0.90% | 1.68% |  |
