# Supplementary material for: Non-destructive Plant Morphometric and Color Analyses Using an Optoelectronic 3D Color Microscope
Source: Front Plant Sci. 2018 Sep 25;9:1409. doi: 10.3389/fpls.2018.01409 (PMC6167917; doi:10.3389/fpls.2018.01409)
Supplement: Supplementary file 4 [file Table_4.DOCX]

**Supplementary Table S4.** Manual measurements acquired from 5 independent reconstructions of the 1x1 matt painted LEGO piece shown in Figure 8F and Supplementary Figure S5B and C.

| **3D Reconstruction** | **length** | **width** | **height** |
| --- | --- | --- | --- |
| **1** | 7421 | 7415 | 11757 |
| **2** | 7467 | 7376 | 11756 |
| **3** | 7443 | 7379 | 11758 |
| **4** | 7456 | 7442 | 11768 |
| **5** | 7464 | 7434 | 11779 |
| **average** | 7450 | 7409 | 11763 |
| **standard deviation** | 18.8 | 30.6 | 9.9 |
