# Supplementary figures and images for: Non-destructive Plant Morphometric and Color Analyses Using an Optoelectronic 3D Color Microscope
Source: Front Plant Sci. 2018 Sep 25;9:1409. doi: 10.3389/fpls.2018.01409 (PMC6167917; doi:10.3389/fpls.2018.01409)

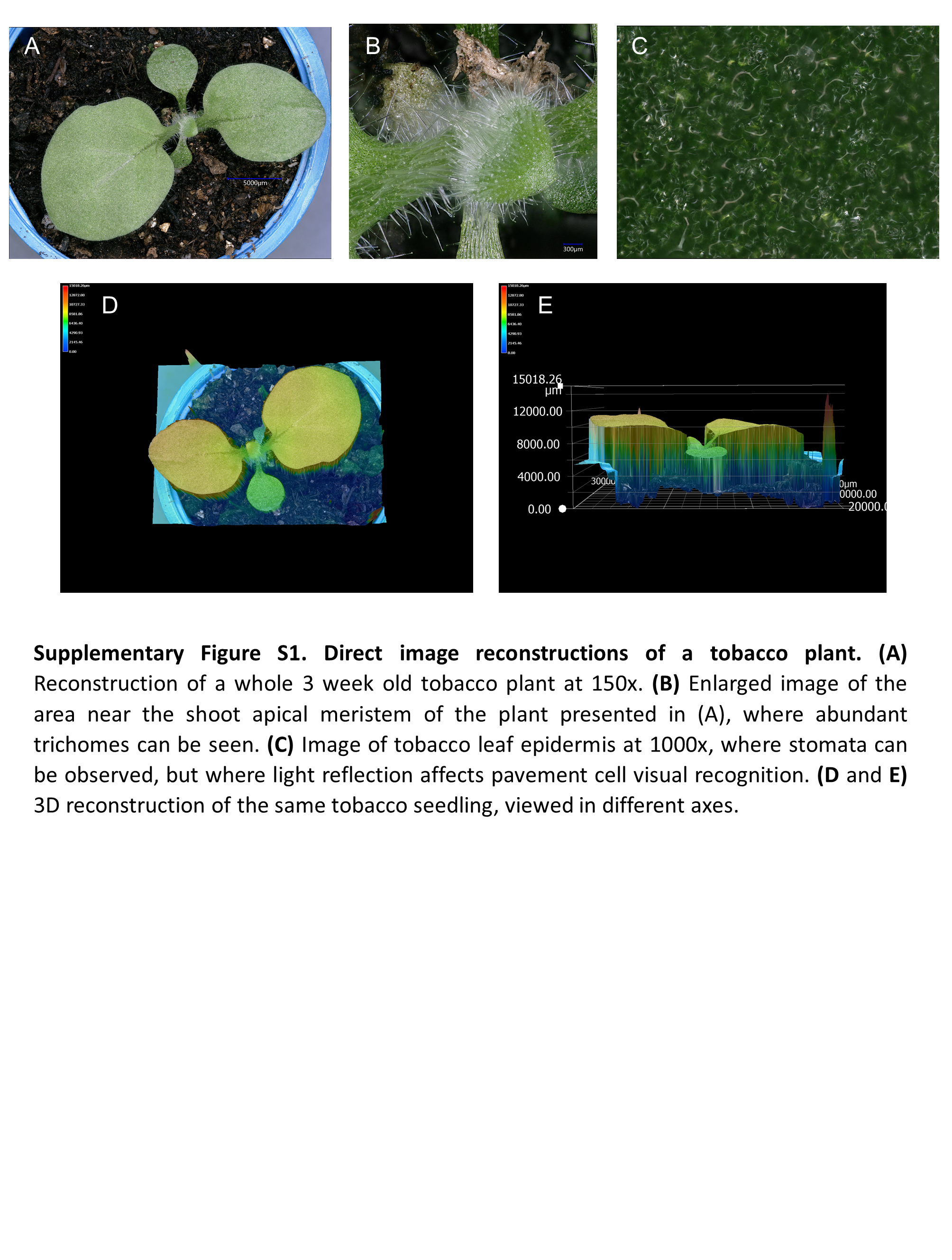

Supplement: Supplementary file 6 [file Image_1.TIF]

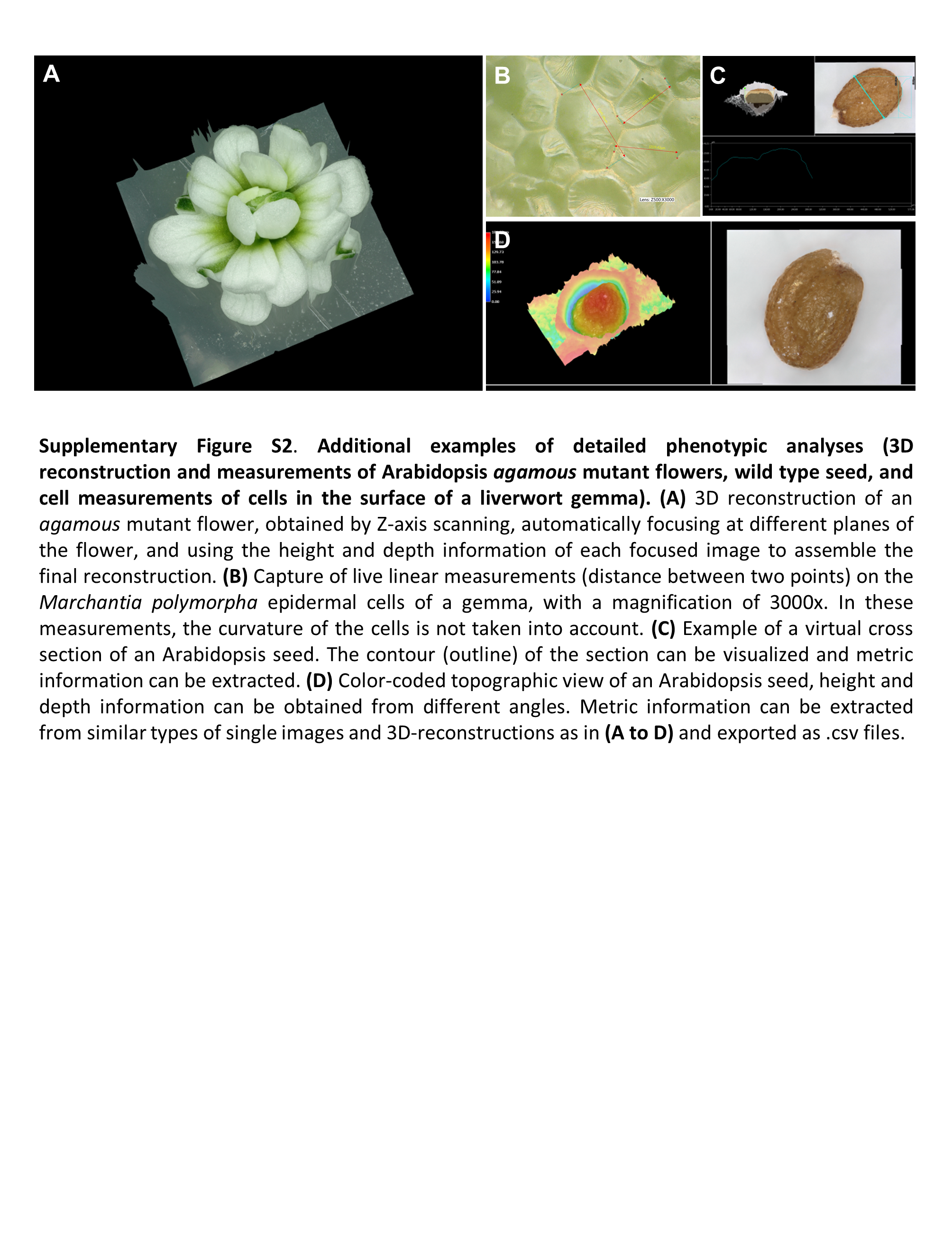

Supplement: Supplementary file 7 [file Image_2.TIF]

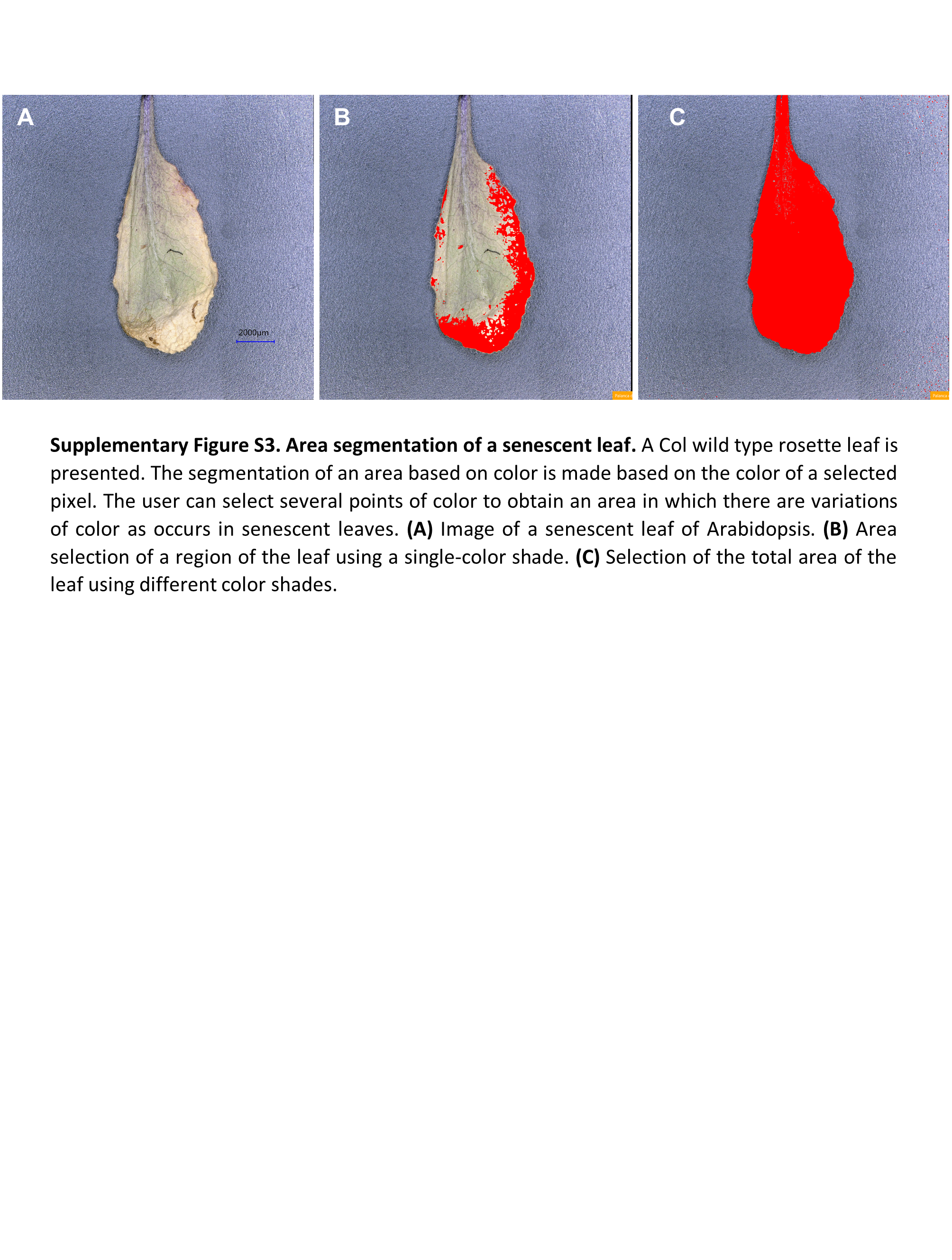

Supplement: Supplementary file 8 [file Image_3.TIF]

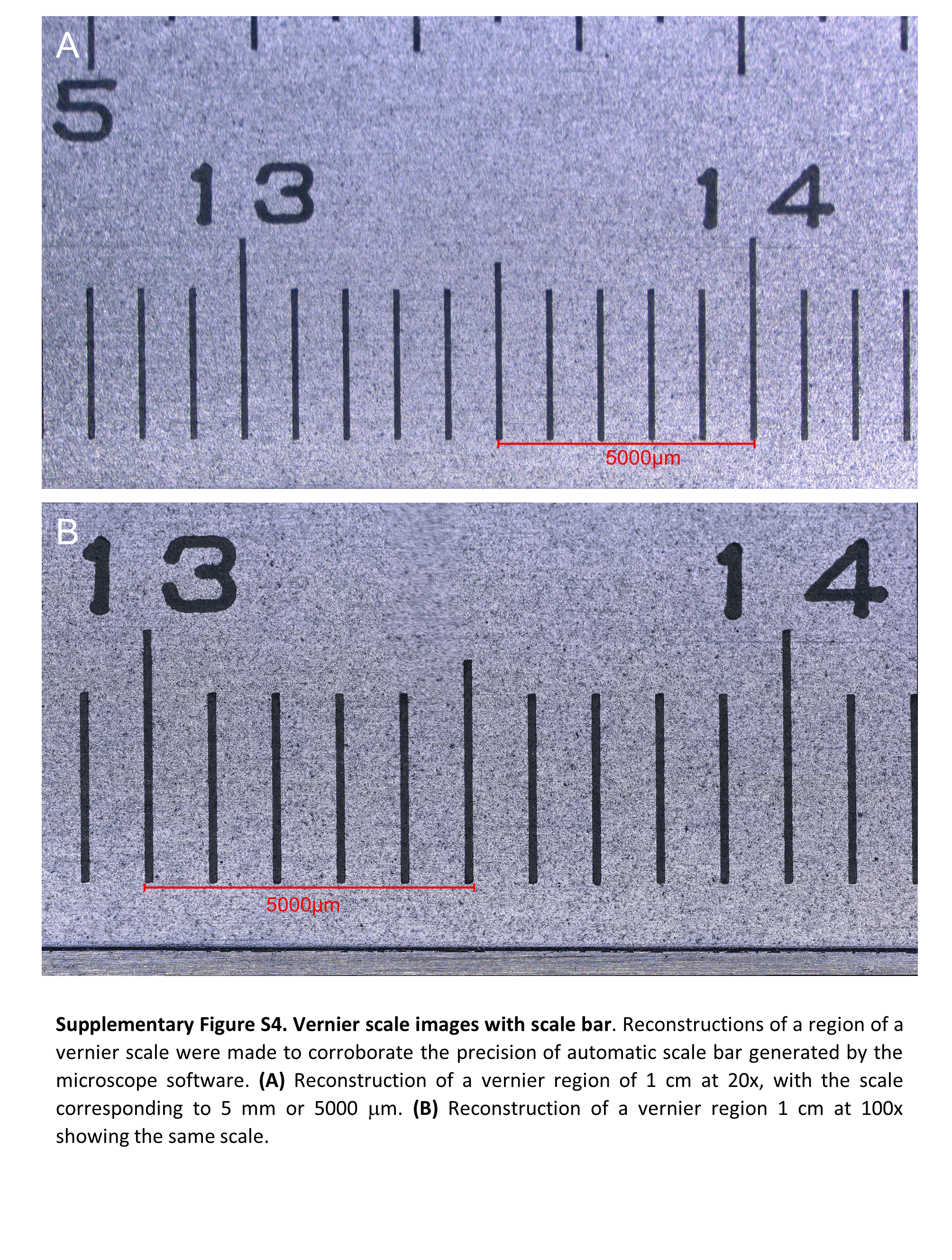

Supplement: Supplementary file 9 [file Image_4.TIF]

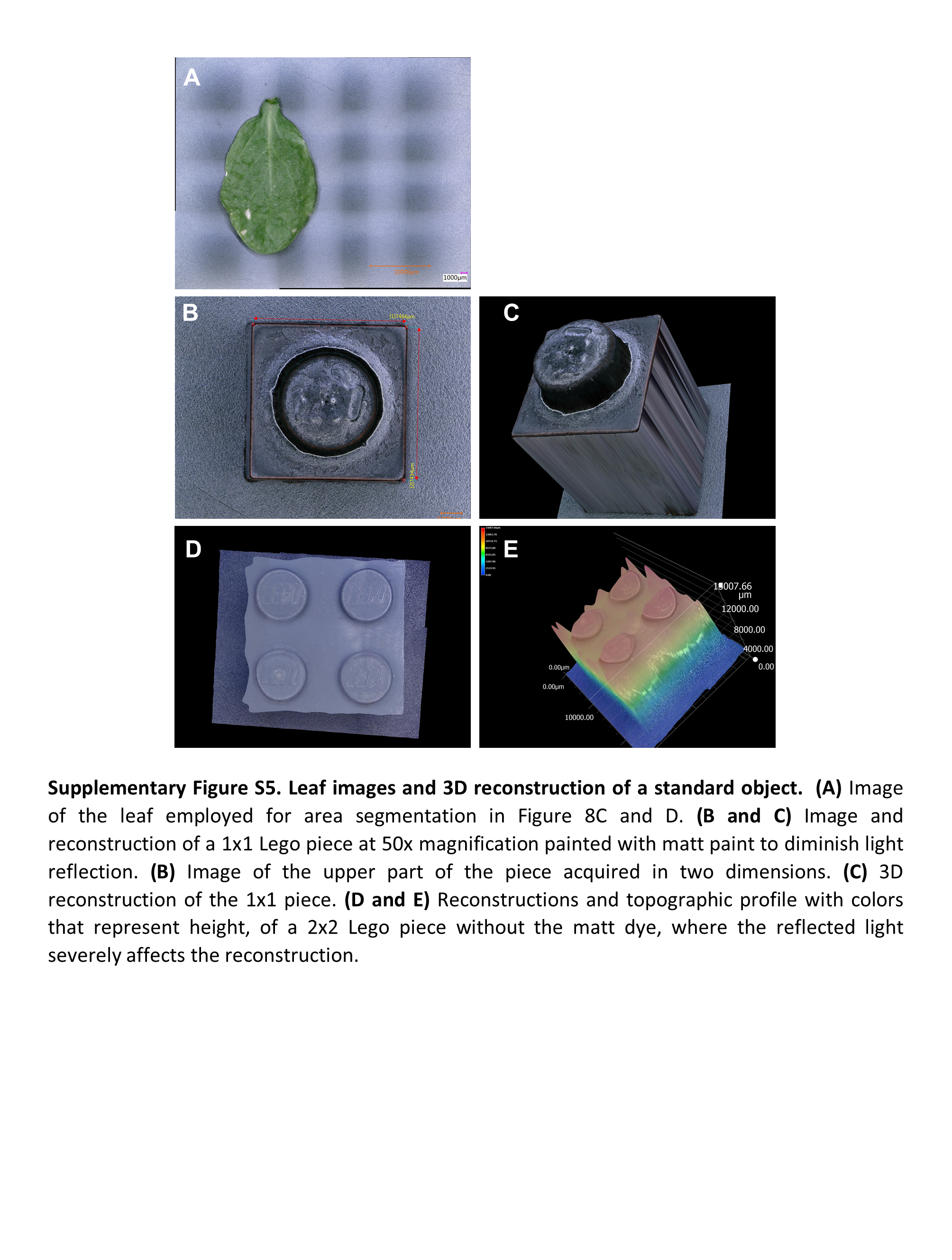

Supplement: Supplementary file 10 [file Image_5.TIF]

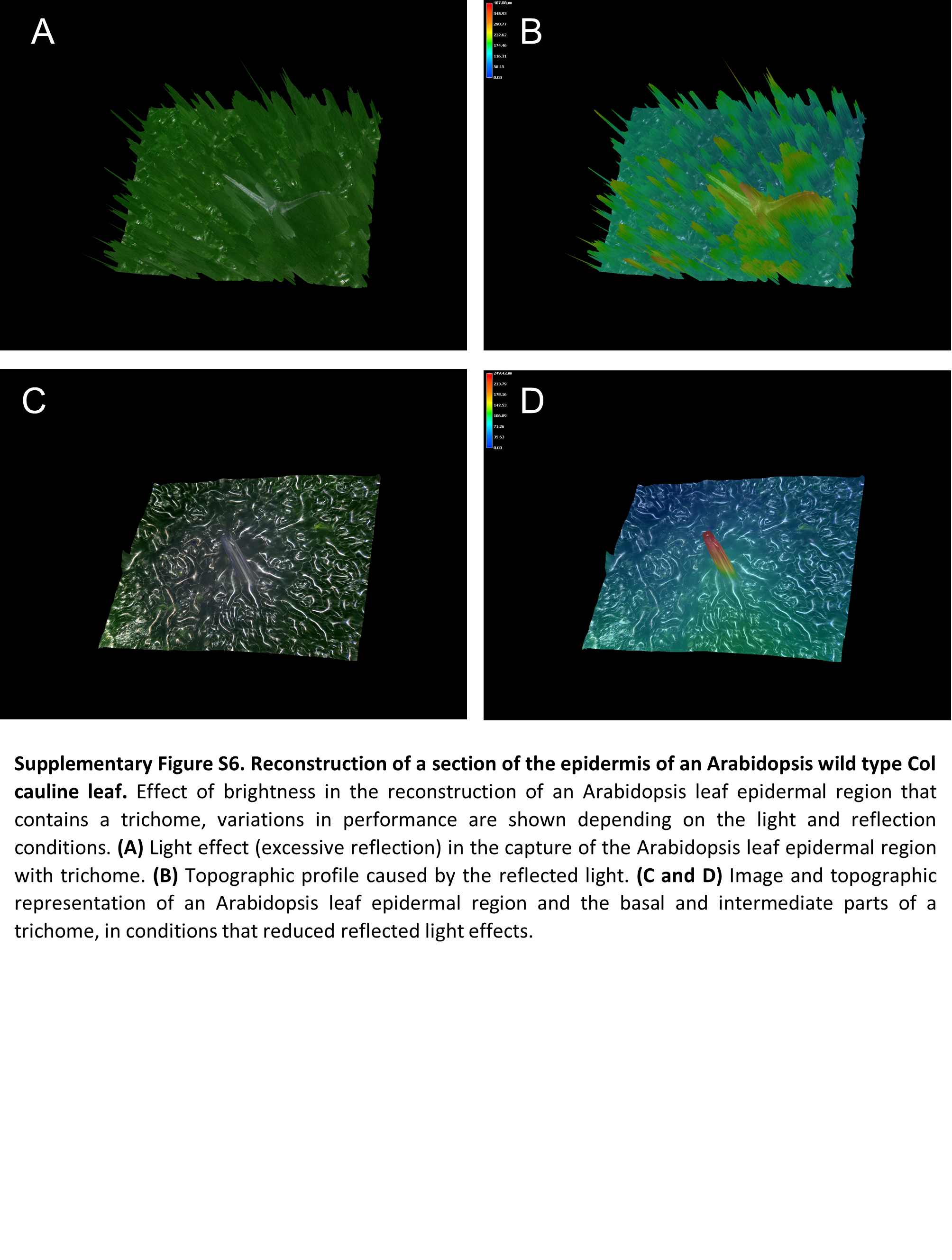

Supplement: Supplementary file 11 [file Image_6.TIF]
